# Essential and Nonessential Elements, Lipids and Volatile Compounds in Coffee and Transfer to Coffee Brews: Assessment of the Benefits and Potential Risks for Human Health

**DOI:** 10.1002/fsn3.4640

**Published:** 2024-12-02

**Authors:** Marin Senila, Eniko Kovacs, Lacrimioara Senila

**Affiliations:** ^1^ INCDO INOE 2000, Research Institute for Analytical Instrumentation Cluj‐Napoca Romania

**Keywords:** dietary intake, metals, fatty acids, volatiles, risk assessment, trace elements

## Abstract

Coffee is a popular beverage with significant commercial and social importance. The study aimed to determine the fatty acids profile, volatile compounds, and concentration of major and trace elements (Na, Mg, K, Ca, P, S, Fe, Mn, Cu, Zn, Cr, Ni, Cd, and Pb) in the two most important varieties of coffee, namely arabica and robusta. The leaching percentages of mineral elements and the effect of boiling time on the transfer of elements to aqueous extracts were also determined. In terms of fatty acids profile, the robusta variety was found to have a higher content of saturated fatty acids (46.68%) compared to the arabica variety (44.38%), whereas arabica contained a higher amount of omega‐6. Regarding the volatile compounds, arabica contained higher amounts of pyrazines (36%), ketones (5.4%), and furans (37.8%). The average contents of the major elements in roasted coffee (arabica and robusta) decreased in the order K > P > S > Mg > Ca > Na, while the trace elements content exhibited a decrease in the order Fe > Mn > B > Cu > Zn > Ni > Pb > Cd ≈ Cr. In coffee brews, the contents of elements have a similar decreasing trend, although variations in leaching percentages were observed. The health risk was assessed considering the concentrations of potentially toxic elements in coffee brews, and no health risks were indicated by the obtained scores. The contribution of coffee brews to the recommended dietary daily intake of essential elements is low. However, it can be significant considering that this beverage is consumed on a daily basis.

## Graphical abstract

Mineral compositions, fatty acids, and volatile compounds of coffee were analyzed. Minerals have a variable leaching percentage to solution depending on the element. The contribution of coffee drinks to the recommended dietary intake of minerals is low.

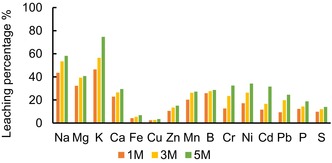

## 1 Introduction

Due to its pleasant flavor and taste, coffee is a very popular brew with an enormous commercial and social importance (Fernandes et al. 2005; Grembecka, Malinowska, and Szefer 2007; Winiarska‐Mieczan et al. 2023). It is estimated that about 40% of the world's population consumes at least one cup of coffee per day (Janda et al. 2020). For example, in the period 2020–2021, the estimated coffee consumption in the world was over 165 million bags (Weinberger et al. 2024). Coffee plants belong to the *Rubiaceae* family, *Coffea* genus, and two principal varieties are of particular importance in marketable trade: *Coffea arabica* , labeled as arabica and *Coffea canephora* labeled as robusta (Da Costa et al. 2024; Muñiz‐Valencia et al. 2014; Valentin and Watling 2013; Vezzulli et al. 2023). Arabica coffee is generally considered to be superior to robusta due to its organoleptic characteristics, the brew resulted from robusta being less aromatic and more bitter (Di Stefano et al. 2023; Habte et al. 2016).

The consumption of moderate quantities of coffee brews can offer a wide range of benefits to human health, such as a reduced risk of developing colon cancer, cirrhosis, or Parkinson's disease (Batista Dos Santos Espinelli Junior et al. 2020; Grembecka, Malinowska, and Szefer 2007; Winiarska‐Mieczan et al. 2023). Mainly due to caffeine (C_8_H_10_N_4_O_2_), an alkaloid present in the coffee brew, coffee is recognized for its ability to reduce fatigue, improve mental concentration, and increase energy and productivity (Debastiani et al. 2019; Giontella et al. 2023; van der Linden, Olthof, and Wijnhoven 2023). Other beneficial properties of coffee are derived from a number of compounds extracted from roasted coffee and dissolved in hot water, including carbohydrates, lipids, phenols, vitamins, alkaloids, and minerals (Cordoba et al. 2020). Some possible negative effects of excessive coffee consumption are increased blood pressure or a reduced adsorbtion of Ca (Samsonowicz et al. 2019).

Coffee beans contain minerals, among which essential elements (K, Ca, P, Fe, Mn, Zn, and Cu) with a positive effect on the human health (Albals et al. 2021). Other elements such as Pb, Hg, and As, have no known biological function, being potentially toxic elements (PTEs), and representing a risk to human health, even at low concentrations. Even some essential elements may become toxic at concentrations above certain limits (Ali, Khan, and Ilahi 2019). Six trace elements (Cd, Cu, Cr, Mn, Se, and Zn) are reported to have an intriguing relation with human health, revealing both nutritional and toxicological effects (Filippini et al. 2018). Ingestion of PTEs was found to affect the immunologic, digestive, skeletal, and nervous systems (Atamaleki et al. 2020; Senila 2023). Several studies reported that coffee brew contains essential elements such as K and Mg, as well as some micronutrients (Janda et al. 2020; Olechno et al. 2021; Oliveira et al. 2012).

The concentration of substances such as minerals, lipids, and volatile compounds in coffee beans depends on their concentration in soil, climate, and processing practice (Debastiani et al. 2019). During the coffee drink preparation by direct contact of roasted coffee with hot water, the parts of these compounds are transferred to the brew, in a solid–liquid extraction process which can be modeled based on Fick's law of diffusion (Cordoba et al. 2020). The solubility of minerals in hot water differs, since some of them are more water‐soluble than others (Cruz, Morais, and Casal 2015). Lipids content strongly influences the coffee brew flavor because they produce emulsions that preserve aromatic compounds and enhance the texture perception in coffee (Cordoba et al. 2020). However, only parts of the lipids are emulsified due to their immiscibility with water. Despite the fact that there are several studies in the literature dealing with the components of coffee beans and the influence of the processing methods on the composition (Al‐Jaf and Saydam 2019; Batista Dos Santos Espinelli Junior et al. 2020; Debastiani et al. 2019; Di Stefano et al. 2023; Pohl et al. 2022; Santos et al. 2021; Vezzulli, Lambri, and Bertuzzi 2023), the effect of the percolation time on the transfer of the minerals has not been extensively studied. Very scarce information exists on the extent to which coffee drinks cover the recommended daily intakes (RDIs) of essential minerals (i.e., Fe, K, Ca, Mg, Mn, Cu, and Zn), or on the possible health risks due to potentially toxic elements, as they contain relatively low amounts of minerals, compared with other food components. Nonetheless, since coffee brews are consumed daily, these can bring an important fraction of the necessary daily intake, thus the evaluation of their content is very important. Limited information is also reported on the profile of fatty acids and volatile compounds in coffee and their possible effects on human health, whereas to the best of the authors' knowledge, no data exists on the nutritional quality indices based on the fatty acids profile of coffee.

The first objective of this work was to assess the composition of the two main types of roasted *arabica* and *robusta* coffee in terms of essential and nonessential elements, and their profile of fatty acids and volatile compounds. The second objective was to evaluate the leaching of mineral elements from roasted coffee to coffee brews according to the boiling time, because it is expected that different elements have different extraction degree. We used these data to assess the contribution of coffee brews consumption to the dietary intake and health risks, based on their composition in terms of mineral elements. The third objective of this study was to evaluate the nutritional quality indices based on the fatty acids profile of coffee since this can have effects on human health.

## 2 Materials and Methods

### 2.1 Coffee Samples and Preparation of Coffee Brews

In this study, six roasted coffee types (three arabica and three robusta) with origins from Brazil, Colombia, and Ethiopia, commercially available on the Romanian market, were purchased and analyzed. Each fine ground coffee was used to prepare three boiled coffees in distilled water, by mixing 4 g of fine ground coffee with 100 mL hot water. To assess the influence of boiling time on the extracted minerals from the solid to the water phase, three different boiling times were used: 1, 3, and 5 min. Subsequently, the obtained coffee beverages were immediately filtered through filter paper in other recipients in order to ensure the end of elements transfer after these specific times.

### 2.2 Chemicals, Analytical Instrumentation, and Analytical Methods

#### 2.2.1 Reagents, Standard Solutions, and CRMs

Nitric acid, 60% suprapure, and perhydrol 30% ultrapure were obtained from Merck (Darmstadt, Germany). The calibration standards for metals determination were prepared by appropriate dilutions from the 1000 mg L^−1^ multi‐element IV ICP solution or from single‐element (Cu, Zn, Cr, Ni, Cd, and Pb) ICP standards (1000 mg L^−1^) produced by Merck (Darmstadt, Germany). Matrix modifiers used in GFAAS measurements were prepared from 10% NH_4_H_2_PO_4_, 1% MgNO_3_, and 1% Pd in 10% HNO_3_, purchased from Perkin Elmer (Shelton, CT, USA). An ultrapure water (Elga Veolia, High Wycombe, United Kingdom) system was used for sample preparation. A certified reference material GBW10014 Cabbage (Institute of Geophysical and Geochemical Exploration, Langfang, China) was used to check the accuracy of the element's determination methodology. Chloroform (for analysis EMSURE), methanol (laboratory grade), potassium chloride (for analysis EMSURE), sodium chloride (for analysis EMSURE), sodium hydrogen sulfate monohydrate (extra pure), potassium methoxide (95%), and the standard FAME mixture (Supelco 37 component FAME mix, CRM47885) produced by Sigma‐Aldrich (Merck KGaA, Darmstadt, Germany) were used for the analysis of fatty acids and volatile compounds.

#### 2.2.2 Analysis of the Mineral Composition of Roasted Coffee and Aqueous Extracts

A microwave oven equipped with 12 polytetrafluoroethylene (PTFE) digestion closed vessels (XPERT, Berghof, Eningen, Germany) was used to digest the samples for elemental analysis. The PTFE vessels were pre‐cleaned with 10% (v/v) HNO_3_, then were thoroughly washed with ultrapure water to avoid contamination. A portion of 500 mg of homogenized fine ground coffee was accurately weighed into the PTFE vessel, then 6.0 mL of 60% HNO_3_ and 2.0 mL of 30% H_2_O_2_ were added. The samples were kept in the opened vessels for 2 h at room temperature under a fume hood for predigestion and then the vessels were sealed. A four steps digestion program with a maximum temperature of 220°C and a total time of 45 min was employed. After cooling down to room temperature, the digests were filtered (using a Whatman No. 40 filter), transferred into 25 mL volumetric flasks and diluted to volume with ultrapure water. For the digestion of coffee brews, 20 mL of sample was mixed with 3.0 mL of 60% suprapure HNO_3_ and 2.0 mL of ultrapure 30% H_2_O_2_, then refluxed 2 h on a hotplate, until a clear solution was obtained. The solution was filtered (using a Whatman No. 40 filter) into a 25 mL volumetric flask and diluted to volume with ultrapure water. The filtrates were stored in polyethylene bottles until elemental analysis. The concentrations of major elements (Na, Mg, K, Ca, P, S, Fe, and Mn) were quantified using an inductively coupled plasma optical emission spectrometer (ICP‐OES) Optima 5300 DV Perkin Elmer (Shelton, CT, USA). The concentrations of trace elements (Cu, Zn, Cr, Ni, Cd, and Pb) were measured using a graphite furnace atomic absorption spectrometer (GFAAS) PinAAcle 900T Perkin Elmer (Shelton, CT, USA). Six‐points calibration curves were used for the instrument calibration, over the range of 0–20 mg L^−1^ for all the elements measured by ICP‐OES and 0–50 μg L^−1^ Cu, 0–5 μg L^−1^ Zn, 0–50 μg L^−1^ Cr, 0–50 μg L^−1^ Ni, 0–5 μg L^−1^ Cd, and 0–20 μg L^−1^ Pb for the elements measured by GFAAS. Three replicates were analyzed for the quantification of each element.

#### 2.2.3 Analysis of Fatty Acids Compositions

##### 2.2.3.1 Extraction of Lipids From the Samples of Coffee Beans

The method employed for lipid extraction from coffee samples was conducted in accordance with Arumugam, Baskar, and Sriram (2024), with adjustments. The samples (3 g) were extracted with 50 mL of chloroform: methanol (2:1, v/v), prior to their introduction into an ultrasonic bath (ISOLAB, Eschau, Germany). The extraction was conducted in an ultrasonic bath with the following specifications: 150 × 138 × 65 mm^3^ dimensions, 1.3 L volume, 60 W ultrasonic power, and 40 kHz frequency. The extraction process was repeated four times, with a duration of 15 min each. Following the extraction, the samples were filtered, and the liquid fraction was recovered and extracted with 20 mL of KCl (0.74%). The extracts were centrifuged (10 min at 3075 g force) to separate the organic phase, which was then filtered using Na_2_SO_4_ · H_2_O to eliminate the water. The solvent was evaporated using a rotary evaporator (Laborota 4010, Heidolph, Scwabach, Germany) and the oil obtained was dried at 60°C in an oven.

##### 2.2.3.2 Fatty Acid Methyl Esters (FAMEs)

Fatty acids composition of roasted coffee oil was determined by gas chromatography coupled with flame ionization detector techniques, after transesterification of fatty acids to fatty acid methyl esters with potassium methoxide. The coffee samples (0.06 g) were introduced in isooctane, then 0.2 mL methanolic potassium hydroxide solution (CH_5_KO_2_) 2 mol L^−1^ was added and vigorously stirred for 30 s. Subsequently, the mixture was treated with 1 g of sodium hydrogen sulfate (NaHSO_4_ · H_2_O) to avoid saponification of methyl esters and neutralize excess alkali. Each oil sample was trimethylated and analyzed in three replicates. The results are expressed in relative percentage of each fatty acid, calculated by the internal normalization of the chromatographic peak area.

##### 2.2.3.3 Determination of Fatty Acid Methyl Esters (FAMEs) Content Using GC‐FID

The fatty acid methyl esters (FAMEs) content in coffee beans were determined using a gas chromatograph with flame ionization detector (GC‐FID) (6890N, Agilent Technologies, Santa Clara, CA, USA), equipped with a ZB‐WAX capillary column (30 m × 0.25 mm × 0.25 μm) (Agilent Technologies, Santa Clara, CA, USA). The carrier gas was helium (6.0 purity, Linde Gaz, Cluj‐Napoca, Romania) at a constant flow rate of 1 mL min^−1^. The split ratio was 1:20, and the injected volume was 1 μL. The GC oven temperature program consisted of three stages: 60°C for 1 min, from 60°C to 200°C (rate 10°C min^−1^, 2 min), and from 200°C to 220°C (5°C min^−1^, 20 min). Both injector and detector temperatures were at 250°C to ensure the complete vaporization of the sample and detection sensitivity. The retention times of the sample FAMEs were compared with those of the FAME standard mixture (Supelco 37 component FAME mix, CRM47885).

#### 2.2.4 Estimation of Volatile Compounds

Volatile compounds were determined using a gas chromatograph coupled to a mass spectrometer (GC–MS) (6890N, Agilent Technologies, Santa Clara, CA, USA), equipped with a HP‐5‐MS capillary column (60 m length, 0.2 mm I.D., and 0.25 μm film thickness) (Agilent Technologies, Santa Clara, CA, USA). A quantity of 3 g of coffee sample was transferred to a 20 mL headspace vial and 3 g of NaCl were added to help increasing the volatility of these compounds and to inhibit any enzymatic reactions. The volatile compounds were identified by the NIST mass spectrometry library (NIST 11). The identification of chemicals was determined by matching with NIST 11 at ≥ 70% matching factors. All measurements were conducted in triplicate, and data are presented as the mean ± standard deviation.

### 2.3 Quality Control for Elemental Analysis

The accuracy of the methods for the total concentration of metals in coffee was assessed by analyzing the certified reference material GBW10014 Cabbage (Institute of Geophysical and Geochemical Exploration, Langfang, China). The recoveries were found to be between 87% and 106%, indicating a satisfactory performance.

### 2.4 Assessment of Potential Risk to Human Health Posed by Toxic Elements Extracted From Coffee Brews

The noncarcinogenic risk to human health posed by toxic elements in coffee was evaluated through the application of the hazard quotient (HQ) and hazard index (HI). The HQ associated with each measured heavy metal ingestion through coffee consumption was calculated according to Equation (1) (Senila et al. 2023). HI indicates the overall risk associated with the sum of all analyzed potentially toxic elements (Giri et al. 2021).

(1) $$\mathrm{HQ}=\frac{\mathrm{PTE}\times \mathrm{IR}\times \mathrm{ED}\times \mathrm{EF}}{\mathrm{AT}\times \mathrm{BW}\times R_{f}D}$$

where PTE is the content of each potentially toxic element (mg L^−1^) in the coffee brew and IR represent the average ingestion rate of coffee (L day^−1^). HQ was calculated for a variable ingestion rate of 1–3 cups of coffee per day (one cup being considered as 0.1 L). ED is the exposure duration (56 years) and EF represents the exposure frequency (365 days year^−1^). AT and BW are the average exposure time (365 days year^−1^ × ED) and, respectively, the body weight (70 kg). R_f_D is the reference dose for each ingested element analyzed in samples according to the United States Environmental Protection Agency (USEPA 1993) through the integrated risk information system (IRIS), as 0.001 mg kg^−1^ day^−1^ Cd, 1.5 mg kg^−1^ day^−1^ Cr, 0.04 mg kg^−1^ day^−1^ Cu, 0.7 mg kg^−1^ day^−1^ Fe, 0.0035 mg kg^−1^ day^−1^ Pb, 0.14 mg kg^−1^ day^−1^ Mn, 0.02 mg kg^−1^ day^−1^ Ni, and 0.3 mg kg^−1^ day^−1^ Zn (Atamaleki et al. 2020). The threshold for HQ is 1.0; if HQ < 1.0, there are no noncarcinogenic risk effects, while HQ > 1.0 indicates that the consumed coffee may cause noncarcinogenic risk (Noman et al. 2022).

### 2.5 Assessment of Fatty Acids‐Based Nutritional Quality Indices of Coffee Lipids

Based on the fatty acid profiles of the extracted lipids, the nutritional quality of the coffee brews was evaluated using several indices. These comprised the thrombogenic index (TI), the atherogenic index (AI), the hypocholesterolemic/hypercholesterolemic ratio (h/H), the health‐promoting index (HPI), and the nutritive value index (NVI) (Dongmo et al. 2024). The calculation formulas are revealed in Table 1 (Dongmo et al. 2024; Senila, Senila, and Resz 2024).

**TABLE 1:** Nutritional quality indices and calculation formulas.

Parameters	Calculation formulas
TI^a^	$\frac{\left(C14:0+C16:0+C18:0\right)}{[\left(0.5\times \sum \mathrm{UFA}\right)+\left(0.5\times \sum \text{PUFA}\;n-6\right)+\left(3\times \sum \text{PUFA}\;n-6\right)+\left(\sum \text{PUFA}\;n-3/\sum \text{PUFA}\;n-6\right)]}$
AI^b^	$\frac{\left[C12:0+\left(4\times C14:0\right)+C16:0\right]}{\sum \mathrm{UFA}}$
h/H^c^	$\frac{\left(\mathrm{cis}-C18:1+\sum \text{PUFA}\right)}{\left(C12:0+C14:0+C16:0\right)}$
HPI^d^	$\frac{\left(\sum \mathrm{UFA}\right)}{\left[C12:0+\left(4\times C14:0\right)+C16:0\right]}$
NVI^e^	$\frac{\left(C18:0+C18:1\right)}{C16:1}$

^a^ Thrombogenic index.

^b^ Atherogenic index.

^c^ Hypocholesterolemic/hypercholesterolemic ratio.

^d^ Health‐promoting index.

^e^ Nutritive value index.

### 2.6 Statistical Analyses

The correlation analyses were performed with the Tukey's test (*p* = 0.05) using the Paired Comparison App (Two‐way ANOVA) by Origin software (version 2020b, OriginLab, Northampton, MA, USA). Tukey's test was applied to compare separately the roasted arabica and robusta types, coffee brews from arabica and from robusta types. The different letters indicate statistically significant differences at a level *p* < 0.05.

## 3 Results and Discussion

### 3.1 Content of Mineral Elements in Roasted Coffees

The average contents of each element found in roasted coffee (arabica and robusta) and those extracted in coffee brews after 1, 3, and 5 min boiling time are given in Table 2. The elements with the highest contents in roasted coffee types were K, P, S, Mg, Ca, and Na. In the arabica type, the average contents of these elements were K (30.90 g kg^−1^), P (6.86 g kg^−1^), S (3.09 g kg^−1^), Mg (2.72 g kg^−1^), Ca (0.76 g kg^−1^), and Na (0.198 g kg^−1^). In robusta roasted coffee, the average contents were K (23.70 g kg^−1^), P (7.73 g kg^−1^), S (2.19 g kg^−1^), Mg (2.30 g kg^−1^), Ca (1.46 g kg^−1^), and Na (0.146 g kg^−1^). These contents were in agreement with the contents reported by Debastiani et al. (2019), and with the contents found by Janda et al. (2020), except for P, whose content was about two times lower in the current study. Grembecka, Malinowska, and Szefer (2007) reported a similar content of Mg, but lower contents of K and Ca, and a higher content of Na, nevertheless still in the same order of magnitude with the results obtained in the current study. The microelements average contents (mg kg^−1^) in arabica roasted coffee decreased in the order Fe (55.6) > Mn (40.2) > B (32.5) > Cu (24.7) > Zn (8.13) > Ni (1.36) > Pb (0.72) > Cd (0.50) ≈ Cr (0.46). A comparable decreasing trend of microelements content (mg kg^−1^) was observed in robusta coffee: Fe (61.8) > B (33.3) > Cu (27.4) ≈ Mn (26.9) > Zn (7.97) > Ni (2.64) > Pb (0.68) > Cr (0.50) ≈ Cd (0.45). Grembecka, Malinowska, and Szefer (2007) reported contents generally of a similar order of magnitude with those observed in the present study: 5.3 mg kg^−1^ Zn, 16.1 mg kg^−1^ Cu, 41.6 mg kg^−1^ Fe, 22.4 mg kg^−1^ Mn, 0.3 mg kg^−1^ Cr, and 1.6 mg kg^−1^ Ni, while Cd and Pb were below 0.03 and, respectively, below 0.1 mg kg^−1^. In general, good agreements were observed between our results and the contents reported in literature for roasted coffee, by Albals et al. (2021) for Cu, Zn, Pb, Mn, and Fe, by Debastiani et al. (2019) for Cu, Zn, Mn, and Fe, by Jarošová, Milde, and Kuba (2014) for Cu, Fe, Zn, Cd, Cr, Mn, Ni, and Pb, and with the results summarized by Cruz, Morais, and Casal (2015) for Fe, Mn, Cu, and Zn. Also, Khaneghah et al. (2022) conducted a systematic review on PTEs concentrations in coffee products analyzed during the period 2010–2021. According to their performed meta‐analysis, the pooled average content of essential elements is in general considerably higher than that of PTEs, which is in line with the findings of the present study.

**TABLE 2:** The average contents of mineral elements in roasted coffee (mg kg^−1^) and in aqueous extracts (1, 3, and 5 min boiling time) presented in μg L^−1^ (average ± SD).

Element	Arabica roasted (mg kg^−1^ dw)	Robusta roasted (mg kg^−1^ dw)	Coffee brews from arabica (μg L^−1^)			Coffee brews from robusta (μg L^−1^)		
			1 min	3 min	5 min	1 min	3 min	5 min
Na	197 ± 12^a^	146 ± 11^b^	3440 ± 614^a^	4220 ± 51^ab^	4600 ± 185^a^	2030 ± 122^b^	2180 ± 47^b^	2730 ± 91^a^
Mg	2720 ± 70^a^	2300 ± 152^b^	35,170 ± 2660^b^	42,870 ± 1420^a^	44,400 ± 1180^a^	31,500 ± 755^b^	35,500 ± 709^a^	36,800 ± 404^a^
K	30,900 ± 1500^a^	23,700 ± 1660^b^	574,000 ± 13,100^c^	697,300 ± 46,600^b^	921,300 ± 23,300^a^	605,000 ± 7200^c^	733,000 ± 8500^b^	922,300 ± 20,800^a^
Ca	1570 ± 90^a^	1460 ± 46^b^	14,300 ± 610^b^	16,600 ± 1150^ab^	18,440 ± 945^a^	11,900 ± 208^b^	13,700 ± 250^a^	14,000 ± 210^a^
P	6860 ± 210^b^	7730 ± 260^a^	33,600 ± 557^c^	39,400 ± 669^b^	51,400 ± 2460^a^	42,900 ± 1090^c^	47,700 ± 1060^b^	68,000 ± 1870^a^
S	3090 ± 280^a^	2190 ± 198^b^	12,100 ± 687^c^	14,700 ± 429^b^	17,300 ± 557^a^	13,400 ± 900^b^	16,200 ± 755^ab^	18,300 ± 1700^a^
Fe	55.6 ± 5.1^b^	61.8 ± 4.0^a^	94.0 ± 7.5^c^	120 ± 6.9^b^	150 ± 11.1^a^	99.3 ± 8.4^b^	122 ± 3.2^a^	130 ± 7.2^a^
Cu	24.7 ± 1.8^b^	27.4 ± 1.6^a^	23.7 ± 2.3^b^	26.0 ± 2.0^b^	34.0 ± 2.6^a^	17.7 ± 1.5^b^	22.3 ± 1.5^a^	24.7 ± 1.6^a^
Zn	8.13 ± 0.22^a^	7.97 ± 0.39^a^	34.3 ± 7.5^b^	43.7 ± 5.7^ab^	49.0 ± 2.6^a^	26.0 ± 2.0^b^	35.0 ± 2.0^a^	36.7 ± 2.5^a^
Mn	40.2 ± 2.6^a^	26.9 ± 2.9^b^	326 ± 10^b^	421 ± 10^a^	437 ± 13^a^	184 ± 10^b^	207 ± 7^a^	224 ± 9^a^
B	32.5 ± 1.2^a^	33.3 ± 1.9^a^	336 ± 31^a^	358 ± 10 ^ab^	373 ± 20^bc^	295 ± 6^c^	354 ± 11^b^	385 ± 7^a^
Cr	0.46 ± 0.10^a^	0.50 ± 0.08^a^	2.3 ± 0.6^a^	4.3 ± 0.6^a^	6.0 ± 1.0^a^	1.3 ± 0.6^b^	3.7 ± 0.6^b^	6.3 ± 1.5^a^
Ni	1.36 ± 0.15^b^	2.64 ± 0.29^a^	9.3 ± 0.6^c^	14.3 ± 1.5^b^	18.7 ± 2.1^a^	8.7 ± 0.6^c^	17.3 ± 1.5^b^	22.7 ± 2.1^a^
Cd	0.50 ± 0.07^a^	0.45 ± 0.04^a^	2.3 ± 0.6^b^	3.3 ± 0.6^b^	6.3 ± 1.2^a^	1.7 ± 0.6^b^	3.0 ± 1.0^b^	5.3 ± 0.6^a^
Pb	0.72 ± 0.07^a^	0.68 ± 0.07^a^	2.7 ± 0.6^b^	5.7 ± 0.6^a^	7.0 ± 1.0^a^	3.7 ± 0.6^b^	4.7 ± 0.6^ab^	5.7 ± 0.6^a^

*Note:* Different letters (a, b, and c) in the same row of the same element indicate statistical differences (*p* < 0.05). Statistical comparisons were made separately between elements content in roasted arabica and robusta coffee; contents of elements in aqueous extracts (1, 3, and 5 min boiling time) from arabica, and, respectively, contents of elements in aqueous extracts (1, 3, and 5 min boiling time) from robusta.

### 3.2 Content of Mineral Elements in Coffee Brews and Leaching Percentages

The elements with the highest contents extracted from coffee brews were, like in the roasted coffee, K, Mg, P, S, Ca, and Na. As shown in Table 2, the quantity of extracted elements generally increased in time during the 5 min boiling time.

**FIGURE 1:**
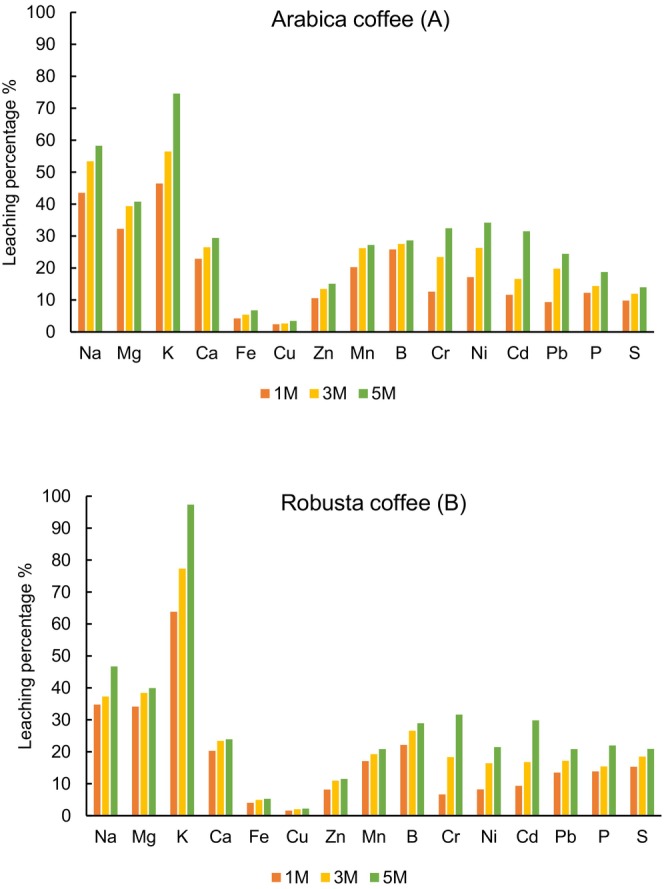
Leaching percentages of mineral elements from roasted coffee (A—arabica, B—robusta) to coffee brews after 1, 3, and 5 min boiling time.

In brews prepared from arabica coffees, the content of K significantly increased from 574 mg L^−1^ after 1 min boiling time to 921 mg L^−1^ after 5 min boiling time. Also, the K content increased from 605 mg L^−1^ after 1 min boiling time to 922 mg L^−1^ after 5 min boiling time in brews prepared from robusta coffees. Considering the amount of coffee and water used to prepare the brews, the extraction percentage from the two coffee types was calculated, at the three percolation times (1, 3, and 5 min), which are displayed in Figure 1. The leaching percentage of K increased from 46% to 75% in the case of arabica type and from 64% to 97% in the case of robusta type. The content of Mg in brews from arabica coffee ranged between 35.2 and 44.4 mg L^−1^, while brews extracted from robusta coffee exhibited concentrations between 31.5 and 36.8 mg L^−1^. This indicates that leaching percentages ranged from 32% to 41% in arabica and, respectively, 34% to 40% in robusta. In the case of Ca, the leaching rate was between 23%–29% (14.3–18.4 mg L^−1^) in the arabica type and 36%–40% (11.9–14.0 mg L^−1^) in robusta coffee. The leaching percentage of Na ranged between 44% and 58% (3.44–4.60 mg L^−1^) in arabica and between 35% and 47% (2.06–2.73 mg L^−1^) in robusta. The other two major elements, P and S, have similar leaching percentages: 12%–19%, and 10%–14% in arabica, and 14%–22%, and respectively, 15%–21% in robusta. These results are comparable to the findings of Grembecka, Malinowska, and Szefer (2007). They reported for major elements average leaching percentages of 75.8% for K, 55.1% for Mg, 47.7% for Na, 30.6% for Ca, and 46.1% for P, respectively. However, Özdestan (2014) reported much lower leaching rates in Turkish coffee samples of only 6.16% for K, and 7.11% for Mg, while Na leaching percentage of 34.31% was of an order of magnitude similar to the results obtained in the current study.

Elements found in trace levels in ground coffee have lower leachabilities than major elements: 2%–3% for Cu, 4%–7% for Fe, 8%–15% for Zn, 7%–32% for Cr, 9%–21% for Pb, 9%–32% for Cd, 8%–34% for Ni, 17%–27% for Mn, and 22%–29% for B. Our results fit well with the average leaching values reported by Grembecka, Malinowska, and Szefer (2007): 3.28% for Cu, 7.80% for Fe, and 24.5% for Mn, respectively. A slightly higher leaching percentages were reported by Grembecka, Malinowska, and Szefer (2007) in the case of Zn (28.7%), Cr (62.2%), and Ni (41.6%). Özdestan (2014) reported for Zn and Mn leaching rates of only 0.7% and, respectively, 5.61% in Turkish coffee.

The low leaching percentages of these trace elements can be attributed to their forms in roasted coffee which render them not completely soluble through an extraction in hot water. For example, Cu, which has the lowest extraction degree, is well‐known for its capacity to form complexes with organic ligands (Senila et al. 2024). The low percolation rates and relatively low contents of these elements in roasted coffee explain the low contents of these elements in coffee brews.

Because no maximum admitted levels are established for metals in coffee brews, the concentrations of elements in coffee were compared with those for drinking water specified in the Directive (EU) 2020/2184 (EU 2020): 5 μg L^−1^ Cd, 10 μg L^−1^ Pb, 20 μg L^−1^ Ni, 25 μg L^−1^ Cr, 200 μg L^−1^ Fe, 50 μg L^−1^ Mn, 2 mg L^−1^ Cu, and 200 mg L^−1^ Na. In general, the concentration of elements in coffee brews were well below the maximum admitted levels (MALs), except for Mn of which concentration exceeded the MAL by about four times in coffees prepared from robusta types, and by about eight times in coffees prepared from arabica types. However, it should be considered that a lower amount of coffee brew is consumed compared to the amount of drinking water recommended for daily consumption (2 L day^−1^).

### 3.3 Assessment of Health Risks due to Ingestion of Coffee Brews

**FIGURE 2:**
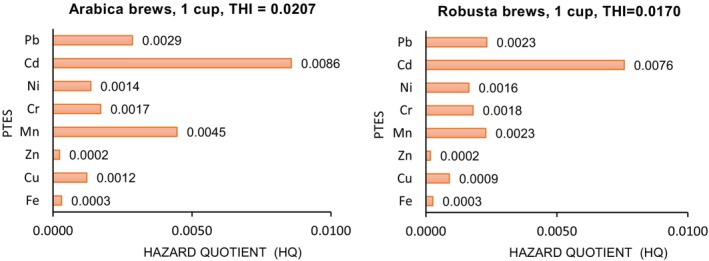
Contribution of each PTE on the hazard quotient, HQ, and THI.

Noncarcinogenic risk to the human health is caused by elements like Cd, Cr, Cu, Fe, Pb, Mn, Ni, and Zn. For the calculation, we used the concentrations of PTEs extracted in coffee brews after 5 min of boiling, having the highest leaching percentages. The hazard quotient (HQ) and the total hazard index (THI) are presented in Figure 2.

As shown in Figure 2, for brews prepared from the arabica types, *HQ* indices ranged between 2.0 × 10^−4^ and 8.6 × 10^−3^, whereas for brews prepared from the robusta types, *HQ* were in the range of 2.0 × 10^−4^—7.6 × 10^−3^. In both cases, scores were much lower than the threshold limit of 1.0, revealing that there are no noncarcinogenic risks related to the drinking of one cup of coffee. Even if 2–3 cups of coffee are consumed, no health risk occurs for the consumers. The *HQ* decreased in the order Cd > Mn > Pb > Cr > Ni > Cu > Fe > Zn in the case of arabica, and almost similar Cd > Mn ≈ Pb > Cr > Ni > Cu > Fe > Zn in the case of robusta type. The scores of THI of 0.0207 in the case of arabica and 0.0170 in the case of robusta were also much lower than 1, demonstrating no health risk for the consumers.

Our results corroborate those of several previous studies in the existing literature. For example, in a recent study on the Cd and Pb concentration in coffee drinks and coffee substitutes, Winiarska‐Mieczan et al. (2023) revealed a very low risk on human health linked to the exposure to Cd and Pb ingested with coffee drinks, with TQH and HI much lower than 1. Taghizadeh et al. (2023) also evaluated the risk of exposure to metals via the consumption of coffee and tea from the Iranian market and reported HQs < 1, at an order of magnitude similar to our results.

### 3.4 Assessment of Contribution to Recommended Dietary Intake

The contribution to recommended dietary intakes of essential elements estimated through the consumption of two cups of 100 mL coffee (prepared from 4 × 2 g of roasted coffee) per day is presented in Table 3. Daily mineral intakes (DMI, %) were determined as DMI = (C/RDA) × 100, where C is the essential element concentration (in mg) in 200 mL coffee brew (extracted from 8 g of coffee during 5 min of boiling), and RDA represents the recommended daily allowance (Şemen et al. 2017).

**TABLE 3:** Estimated dietary mineral intakes (DMI, %) according to reference values of each element by the consumption of 200 mL of coffee brew per day.

Element	RDA mg/day		Arabica, DMI (%)		Robusta DMI, (%)	
	Women	Man	Women	Man	Women	Man
Ca	1000	1000	0.30	0.30	0.28	0.28
Cu	0.9	0.9	0.69	0.69	0.51	0.51
Fe	18	10	0.16	0.30	0.14	0.24
K	4700	4700	3.88	3.88	3.90	3.90
Mg	310	400	2.80	2.17	2.30	1.78
Na	1500	1500	0.06	0.06	0.04	0.04
Zn	8	11	0.09	0.06	0.12	0.09

Considering the average amount of each essential element extracted from 8 g of roasted coffee and the RDA (recommended daily allowance) (Koch et al. 2016), it was calculated that the consumption of 200 mL coffee brew supplies to the human body 0.28%–0.30% of RDA for Ca, 0.51%–0.69% for Cu, 0.14%–0.30% for Fe, 3.88%–3.90% for K, 1.78%–2.80% for Mg, 0.04%–0.06% for Na, and 0.06%–0.12% for Zn. Indeed, this is a low percentage of contribution to the RDA, and it is consistent with the findings of other studies (Grembecka, Malinowska, and Szefer 2007; Koch et al. 2016; Şemen et al. 2017). However, since coffee beverages are consumed daily by most people, its contribution to the human body nutritional requirements can be significant over time.

### 3.5 Lipids Content, Fatty Acids Profiles, and Nutritional Quality Indices

The average lipid content found in the arabica variety was 12.3% ± 1.8%, while in the robusta, it was 10.5% ± 1.4%. The fatty acids composition of arabica and robusta coffees is shown Table 4. According to Zhu et al. (2021), the lipid content in arabica coffee beans plays a significant role in defining their flavor, aroma, and overall quality.

**TABLE 4:** The ranges and concentrations of fatty acids (average ± SD) determined in arabica and robusta coffee varieties presented as % of total acid content.

Type of acids		Arabica	Robusta
Myristic acid	C14:0	0.35 ± 0.03^b^	0.88 ± 0.07^a^
Myristoleic acid	C14:1(n9)	ND	0.48 ± 0.02^a^
Pentadecanoic acid	C15:0	0.57 ± 0.04^a^	0.42 ± 0.03^b^
Pentadesenoic acid	C15:1	0.31 ± 0.02^b^	2.41 ± 0.18^a^
Palmitic acid	C16:0	34.46 ± 1.8^a^	38.22 ± 1.5^a^
Palmitoleic acid	C16:1(n7)	0.04 ± 0.002^b^	0.72 ± 0.02^a^
Heptadecanoic acid	C17:0	0.13 ± 0.01^b^	0.33 ± 0.02^a^
cis‐10‐heptadecenoic acid	C17:1	0.21 ± 0.01^b^	0.32 ± 0.01^a^
Stearic acid	C18:0	6.53 ± 0.4^a^	5.90 ± 0.32^a^
cis + trans‐oleic acid	C18:1(c + t)(n9)	8.29 ± 0.7^a^	6.02 ± 0.4^b^
cis + trans‐ linoleic acid	C18:2(c + t)(n6)	38.53 ± 2.1^a^	30.90 ± 2.6^a^
α‐linolenic acid	C18:3(n6)	0.51 ± 0.04^a^	0.42 ± 0.03^a^
α‐linolenic acid	C18:3(n3)	0.97 ± 0.05^a^	0.26 ± 0.02^b^
Arachidic acid	C20:0	2.34 ± 0.15^b^	0.94 ± 0.08^a^
Gondoic acid	C20:1(n9)	0.31 ± 0.03^b^	0.71 ± 0.07^a^
Eicosadienoic acid	C20:2(n6)	0.21 ± 0.02^b^	0.62 ± 0.07^a^
Heneicosanoic acid	C21:0	0.65 ± 0.04^a^	0.23 ± 0.02^b^
cis‐11,14,17‐eicosatrienoic acid	C20:3(n3)	0.05 ± 0.004^b^	0.53 ± 0.05^a^
Eicosapentaenoic acid	C20:5(n3)	0.02 ± 0.002^b^	0.45 ± 0.06^a^
Behenic acid	C22:0	0.42 ± 0.03^a^	0.21 ± 0.02^b^
Erucic acid	C22:1(n9)	0.89 ± 0.08^b^	6.85 ± 0.52^a^
Docosadienoic acid	C22:2(n6)	1.67 ± 0.12^a^	0.44 ± 0.03^b^
Lignoceric acid	C24:0	2.32 ± 0.18^a^	1.67 ± 0.16^b^
Saturated fatty acids	SFA	44.38 ± 2.5^a^	46.68 ± 3.2^a^
Monounsaturated fatty acids	MUFA	10.06 ± 1.2^b^	17.50 ± 1.1^a^
Polyunsaturated fatty acids	PUFA	41.96 ± 2.1^a^	35.29 ± 2.8^a^
	Omega 6	40.92 ± 2.5^a^	32.39 ± 1.8^b^
	Omega 3	3.36 ± 0.25^a^	2.90 ± 0.24^a^

*Note:* The different letters (a, b) indicate significant differences (*p* < 0.05) between the average results for each component.

The fatty acid composition of coffee beans, particularly the differences between arabica and robusta, can be summarized based on the identified fatty acid methyl esters (FAMEs). The saturated fatty acids found in both coffee varieties are myristic acid (C14:0), palmitic acid (C16:0), margaric acid (C17:0), stearic acid (C18:0), arachidic acid (C20:0), behenic acid (C22:0), and lignoceric acid (C24:0). The fatty acid composition of arabica and robusta coffee varieties exhibited significant differences. The fatty acids that differentiate the two varieties are palmitic acid (C16:0), stearic acid (C18:0), oleic acid (C18:1), and linoleic acid (C18:2). Palmitic acid has a higher content in robusta (38.22%) than in arabica (34.46%), contributing to the stronger flavor of robusta. With regard to stearic acid, the content was similar in both types. The oleic acid (C18:1) content was higher in arabica (8.29%) than in robusta (6.02%), which contributes to the smoother, more delicate flavor of arabica. The linoleic acid (C18:2) content is higher in arabica (38.53%) than in robusta (30.90%), which contributes to the complexity and richness of arabica's flavor profile. Robusta coffee has higher SFA content (46.68%) compared to arabica coffee (44.38%) whereas robusta coffee has a significantly higher MUFA content (17.50%) than arabica (10.06%). Saturated fats are often associated with increased levels of LDL cholesterol in the blood, which can be a risk factor for heart disease. Robusta has a higher SFAs content, which might be less desirable from a cardiovascular health perspective (Shin et al. 2023). Polyunsaturated fats include both omega‐6 and omega‐3 fatty acids, which are essential fats. Arabica coffee has a higher content of polyunsaturated fatty acids (PUFAs), especially omega‐6.

### 3.6 Nutritional Quality Indices

Nutritional quality indices are shown in Table 5.

**TABLE 5:** Nutritional quality indices of coffee varieties.

Quality indices	Arabica beans	Robusta beans
∑ UFA	52.02	52.79
∑ PUFA(n‐6)	40.92	32.39
∑ PUFA(n‐3)	3.36	2.9
TI	0.24	0.29
AI	2.06	4.17
h/H	1.75	1.71
HPI	71.6	49.5
NVI	370.5	16.6

Abbreviations: AI, Atherogenic index; h/H, Hypocholesterolemic/hypercholesterolemic index; HPI, Health‐promoting index; NVI, nutritive value index; TI, Thrombogenic Index.

Unsaturated fatty acids exert an influence on the atherogenic and thrombogenic indices. The Atherogenic Index (AI) index is 2.06 in arabica coffee and 4.17 in robusta coffee. It is recommended to maintain an AI value below 1 and a Thrombogenic Index (TI) value below 0.5, in order to maintain a healthy diet. A high AI value indicates a potential for atherogenesis (formation of fatty deposits in the arteries). Arabica coffee exhibits a lower AI due to its higher unsaturated fatty acid content compared to the higher saturated fatty acid content observed in robusta coffee. However, both coffee varieties exceed the recommended limit. The TI value is less than 0.5 for both coffee varieties, which is a consequence of a more favorable ratio of unsaturated to saturated fatty acids. Both coffee varieties have high indices that indicate a favorable lipid profile for reducing cholesterol levels. A high HPI value in arabica coffee (71.6) indicates a greater proportion of health‐promoting lipids. The high NVI indices found in arabica (370.5) are due to a better lipid profile. The arabica type generally has a more favorable lipid profile, which determines better indices for cardiovascular health and overall nutritive value. In contrast, robusta coffee scores lower on these lipid‐based nutritional quality indices.

### 3.7 Volatile Content in Coffee Beans

The volatile compounds found in coffee beans, including various aldehydes, ketones, esters, alcohols, and hydrocarbons contribute to the aroma. Aldehydes are significant volatile compounds that play a crucial role in the aroma of roasted coffee. Aldehydes in coffee beans, especially in robusta, form primarily during the roasting process due to the Maillard reaction and Strecker degradation. Amines are organic compounds derived from ammonia and play various roles in the sensory profile of coffee (Caporaso et al. 2018).

Table 6 lists the volatile compounds identified in robusta and arabica coffee, before and after heating treatment. Sixty‐one volatile compounds were identified in coffee varieties. The volatile organic compounds (VOCs) are grouped in ketones, pyrazine, pyridine, aldehydes, hydrocarbons, amines, esters, furans, and acids. The volatile compounds are responsible for coffee flavor notes (Bettaieb et al. 2024).

**TABLE 6:** The volatile compounds found in coffee samples, before and after extract.

Nr. crt.	Retention time	Compound name	Roasted arabica	Arabica coffee brew	Roasted robusta	Robusta coffee brew	Sensory descriptors	Compound class
1.	2.799	Acetic acid	2.204 ± 0.1^a^	0.148 ± 0.02^b^	0.10 ± 0.01^b^	0.030 ± 0.002^b^	Pungent, acidic, cheesy, vinegar	Acid
2.	2.981	3,3‐Dimethyl‐2‐butanone	ND	ND	3.020 ± 0.20	1.410 ± 0.1	Buttery, Creamy	Ketone
3.	2.993	trans‐1‐Ethoxy‐1‐butene	3.906 ± 0.2^c^	0.018 ± 0.001^c^	ND	ND	Fruity, sweet	Ketone
4.	3.256	2‐Ethenyl‐2‐butenal	0.022 ± 0.001^b^	0.022 ± 0.001^b^	0.860 ± 0.04^a^	0.120 ± 0.01^b^	Green, herbaceous	Aldehyde
5.	3.437	2‐Methyl‐1‐buten‐3‐yne	0.582 ± 0.04^b^	0.537 ± 0.05^b^	2.030 ± 0.15^a^	0.140 ± 0.02^c^	Not found	Ketone
6.	3.731	2‐Methyl‐1H‐pyrrole	ND	ND	8.590 ± 0.5^a^	4.930 ± 0.5^b^	Sweet, caramel‐like, and roasty	Pyrrole
7.	3.744	1,3‐Diazine	5.281 ± 0.41^a^	1.275 ± 0.11^b^	ND	ND	Roasted, nutty, and chocolate‐like	Pyridazine
8.	3.907	Pyridine	4.777 ± 0.3^b^	3.418 ± 0.2^b^	19.730 ± 1.1^a^	16.190 ± 1.2^a^	Sour, bitter, roasted	Heterocyclic N
9.	4.215	Pyrazine	4.236 ± 0.3^b^	0.126 ± 0.02^c^	7.967 ± 0.5^a^	0.254 ± 0.02^c^	Earthy, nutty, and woody	Pyrazine
10.	4.495	1,3,5‐Cycloheptatriene	0.158 ± 0.02^b^	0.274 ± 0.03^b^	0.860 ± 0.06^a^	0.020 ± 0.001^c^	Not found	Terpenes
11.	4.807	1‐methyl‐Aziridine	0.744 ± 0.06^b^	0.015 ± 0.001^c^	1.380 ± 0.1^a^	0.040 ± 0.003^c^	Not found	Amine
12.	5.158	1‐Buten‐3‐yne	0.092 ± 0.005^a^	0.010 ± 0.001^c^	0.050 ± 0.004^b^	ND	Not found	Ketone
13.	5.345	2‐methyl‐2‐Butenal	0.105 ± 0.01^b^	ND	0.140 ± 0.02^a^	ND	Nutty, green, herbal, floral and sweet	Aldehyde
14.	5.783	2‐methylpyridine	0.003 ± 0.0001^b^	ND	0.070 ± 0.004^a^	ND	Sweetish	Pyridine
15.	6.077	furan‐2‐carbaldehyde	3.746 ± 0.21^b^	1.943 ± 0.15c	8.700 ± 0.56^a^	1.6 ± 0.15^c^	Almond‐like odor, caramel	Furan
16.	6.478	1‐methylpyrrolidin‐2‐one	ND	ND	0.430 ± 0.03^a^	0.080 ± 0.007^b^	Mild amine odor	Ketone
17.	6.809	2‐Methylpyrazine	16.528 ± 1.2^a^	1.180 ± 1.1^c^	17.000 ± 1.5^a^	5.980 ± 4.2^b^	Nutty, cocoa‐like odor	Pyrazine
18.	7.253	Furfural	19.525 ± 1.2^a^	1.909 ± 0.12^c^	4.680 ± 0.23^b^	0.910 ± 0.08^c^	Sweet, nutty, toasty, caramel and aromatic	Furan
19.	7.772	Silacyclohexa‐2,4‐diene	0.049 ± 0.006^b^	0.045 ± 0.004^b^	0.220 ± 0.02^a^	0.050 ± 0.004^b^	Not found	Amide
20.	8.016	2,3‐Dimethyl‐1H‐pyrrole	0.065 ± 0.005^b^	0.032 ± 0.004^b^	0.550 ± 0.05^a^	0.040 ± 0.003^b^	Aromatic, nutty, spicy, earthy	Pyrrole
21.	8.204	2‐ethylimidazole	0.061 ± 0.007^b^	0.092 ± 0.008^a^	ND	ND	Bread‐like, nutty, spicy, umami	Imidazoles
22.	8.323	Formaldehyde, methyl(2‐propynyl) hydrazone	0.027 ± 0.003^b^	0.007 ± 0.0005^b^	ND	ND	Aromatic	Aldehyde
23.	8.392	4‐Aminopyrimidine	0.033 ± 0.004^b^	0.017 ± 0.002^b^	2.034 ± 0.21^a^	0.220 ± 0.02^b^	Aromatic	Pyridine
24.	8.898	2‐Furanmethanol	7.077 ± 0.6^a^	0.231 ± 0.03^b^	0.580 ± 0.04^b^	0.300 ± 0.04^b^	Sweet, caramel‐like	Furan
25.	9.605	2‐Hexenal	0.08 ± 0.008^a^	0.051 ± 0.006^b^	0.040 ± 0.003^bc^	0.030 ± 0.002^c^	Green, herbal, and fresh notes	Aldehydes
26.	9.755	3‐Methyl‐4‐methylenetetrahydrofuran	0.08 ± 0.007^a^	0.044 ± 0.005^b^	0.070 ± 0.008^a^	0.030 ± 0.004^b^	Aroma, nutty	Furan
27.	9.874	3‐Methylcyclopentanone	0.003 ± 0.0004^bc^	ND	0.010 ± 0.001^ab^	0.010 ± 0.0001^ab^	Minty, Camphoraceous, subtile aromatic	Ketone
28.	9.912	2‐Cyclobutene‐1‐carboxamide	0.006 ± 0.0005^ab^	ND	0.010 ± 0.001^ab^	0.020 ± 0.002^a^	Not found	Amine
29.	10.225	Acetoxyacetone	0.842 ± 0.07^a^	ND	0.060 ± 0.005^b^	0.020 ± 0.001^b^	Fruity, buttery, dairy	Ketone
30.	10.45	Furfurylmethyl sulphide	0.003 ± 0.0002^b^	ND	0.060 ± 0.008^a^	0.010 ± 0.001^b^	Onion, garlic, sulfuraceous	Sulphide
31.	12.614	Furfuryl methyl ketone	2.240 ± 0.2^a^	0.103 ± 0.01^c^	2.650 ± 0.21^a^	1.570 ± 0.11^b^	Sweet, nuttiness	Furan
32.	12.795	2,5‐Dimethylpyrazine	8.414 ± 0.5^a^	5.644 ± 0.42^b^	6.530 ± 0.51^a^	3.660 ± 0.23^b^	Nutty, peanut	Pyrazine
33.	13.002	2‐Ethylpyrazine	4.569 ± 0.51^a^	0.348 ± 0.02^b^	6.000 ± 0.5^a^	1.310 ± 0.12^b^	Earthy, roasted	Pyrazine
34	13.427	1‐Octen‐3‐yne	0.089 ± 0.007^b^	0.042 ± 0.005^c^	0.210 ± 0.02^a^	0.090 ± 0.008^b^	Not found	Ketone
35.	13.615	1,2‐Cyclooctadiene	0.036 ± 0.004^b^	0.007 ± 0.0006^b^	0.210 ± 0.02^a^	0.030 ± 0.002^b^	Not found	Hydrocarbon
36.	13.978	N‐Methyl‐4‐pyridinamine	0.007 ± 0.0006^b^	0.004 ± 0.0003^b^	0.270 ± 0.02^a^	0.010 ± 0.001^b^	Not found	Pyridine
37.	14.716	Cyclooctane	0.003 ± 0.0002^b^	ND	0.120 ± 0.01^a^	0.020 ± 0.002^b^	Not found	Hydrocarbon
38.	14.979	1‐methyl‐7‐oxabicyclo[4.1.0]heptane	0.066 ± 0.007^a^	0.007 ± 0.0006^b^	0.060 ± 0.005^a^	0.020 ± 0.001^b^	Not found	Hydrocarbon
39.	15.241	(2‐Methyl‐1‐propenyl)cyclopentane	0.010 ± 0.001^c^	0.003 ± 0.0002^c^	0.110 ± 0.02^a^	0.030 ± 0.002^b^	Not found	Hydrocarbon
40.	15.529	5‐Methyl‐2‐furancarboxaldehyde	7.429 ± 0.6^a^	1.699 ± 0.2^b^	6.760 ± 0.4^a^	2.520 ± 0.21^b^	Sweet, caramel‐like	Furan
41.	15.861	3‐Methyl‐2‐pentanone	0.421 ± 0.03^b^	0.064 ± 0.005^c^	1.350 ± 0.15^a^	0.350 ± 0.04^b^	Not found	Ketone
42.	16.023	2,3‐Diazabicyclo[2.2.2]oct‐2‐ene	0.419 ± 0.05^a^	0.003 ± 0.0002^c^	0.210 ± 0.02^b^	0.180 ± 0.02^b^	Not found	Amine
43.	16.223	2‐Methoxypyridine	0.005 ± 0.0006^b^	0.008 ± 0.0009^b^	0.050 ± 0.006^a^	0.020 ± 0.003^b^	Sweetish	Pyridine
44.	17.062	1‐Methylpyrrole	0.050 ± 0.006^b^	0.037 ± 0.005^b^	0.110 ± 0.02^a^	0.090 ± 0.008^a^	Earthy, nutty, and roasted notes	Pyrroles
45.	17.249	Furfuryl acetate	1.156 ± 0.1^a^	0.557 ± 0.06^b^	ND	ND	Sweet, fruity, floral	Amine
46.	17.487	2‐Ethyl‐5‐methylpyrazine	2.378 ± 0.3^b^	0.534 ± 0.04^c^	4.220 ± 0.3^a^	1.850 ± 0.2^b^	Nutty, earthy, subtle chocolatey notes	Pyrazine
47.	17.706	2‐methyl‐5‐hexenenitrile	0.154 ± 0.02^c^	0.024 ± 0.003^c^	6.760 ± 0.6^a^	3.990 ± 0.4^b^	Green, vegetal	Amine
48.	17.869	2‐Ethyl‐6‐methylpyridine	0.009 ± 0.0008^c^	ND	0.270 ± 0.03^a^	0.160 ± 0.02^b^	Tobacco, oak, moss, leather	Pyridine
49.	18.131	2,3‐Butanediimine, N,N′‐difluoro	0.012 ± 0.001b	ND	0.030 ± 0.004^a^	ND	Not found	Amine
50.	18.256	4‐nitrobenzenesulfonic acid	0.159 ± 0.02^a^	0.025 ± 0.003^c^	0.150 ± 0.02^a^	0.060 ± 0.007^b^	Astringent	Acid
51.	18.344	Benzenesulfonic acid, 4‐nitro‐, 2‐aminophenyl ester	0.035 ± 0.004^b^	0.007 ± 0.0008^c^	0.070 ± 0.008^a^	0.030 ± 0.004^b^	Not found	Ester
52.	18.55	2‐Propen‐1‐amine	0.009 ± 0.0008^b^	0.003 ± 0.0004^b^	0.060 ± 0.007^a^	0.020 ± 0.003^b^	Pungent, ammonia‐like	Amine
53.	18.876	2‐methoxy‐ Pyridine	0.009 ± 0.0008^c^	0.007 ± 0.0008^c^	0.130 ± 0.02^a^	0.070 ± 0.008^b^	Sweet, musty, aromatic	Pyridine
54.	19.232	2‐Amino‐1,3‐propanediol	0.031 ± 0.004^a^	0.003 ± 0.0004^b^	ND	ND	Not found	Amino alcohol
55.	19.501	2,3‐dimethyl‐1H‐Pyrrole	0.003 ± 0.0004^a^	ND	ND	ND	Nutty	Pyrroles
56.	20.383	1,4‐Dihydro‐4‐imino‐1‐methylaminopiridine	0.037 ± 0.004^c^	0.007 ± 0.0008^d^	6.760 ± 0.7^a^	0.970 ± 0.08^b^	Nutty, popcorn‐like, caramel‐like aromas	Pyridine
57.	20.521	N,N,2‐Trimethyl‐4‐pyridinamine	0.502 ± 0.05^b^	0.515 ± 0.06^b^	1.340 ± 0.3^a^	0.070 ± 0.008^c^	Not found	Amine
58.	20.69	7‐Methylbicyclo[4.2.0]octa‐1(6),3‐diene‐2,5‐dione	0.034 ± 0.005^c^	0.080 ± 0.009^b^	0.210 ± 0.03^a^	ND	Not found	Ketone
59.	21.734	trans‐3‐Methyl‐1,3,5‐Hexatriene	0.002 ± 0.0003^c^	ND	1.340 ± 0.2^a^	0.030 ± 0.004^b^	Not found	Hydrocarbon
60.	22.035	3‐Methyl‐3‐penten‐1‐yne	0.003 ± 0.0004^c^	ND	0.570 ± 0.06^a^	0.040 ± 0.005^b^	Not found	Hydrazone
61.	23.254	2‐Methyl‐3‐prop‐2‐enoxypyridine	0.008 ± 0.0009^c^	0.006 ± 0.0007^c^	2.290 ± 0.3^a^	0.140 ± 0.02^b^	Not found	Pyridine

*Note:* The values presented indicate the peak area of each compound (%) (average ± SD). The different letters (a, b, c) indicate significant differences (*p* < 0.05) between the average results for each component.

Pyridine, a volatile organic compound (VOC), is a constituent of coffee that contributes to its complex aroma profile. It is formed mainly by the breakdown of trigonelline, an alkaloid present in green coffee beans (Konstantinidis et al. 2023). In this study, pyridine has been detected in high concentrations in robusta coffee (19%) and is present at a lower concentration in arabica coffee (4%). The variety is distinguished by a more pronounced, pungently bitter flavor profile. This is partially attributable to elevated levels of pyridine and related chemical compounds. The roasting process significantly influences the levels of pyridine, which in turn impacts the sensory characteristics of the final coffee product (Caporaso et al. 2018). A total of five pyrazine compounds were identified in both coffee species. These include pyrazine, 2‐methylpyrazine, 2,5‐dimethylpyrazine, 2‐ethylpyrazine, and 2‐ethyl‐5‐methylpyrazine.

The most prevalent pyrazine compound identified in both coffee varieties was 2‐methylpyrazine, which was determined to be approximately 17%. However, the content decreased significantly after the application of a heating pretreatment. The content of 2‐ethylpyrazine was determined to be 6.0% in robusta and 4.56% in arabica, but the content decreased after the application of a heating pretreatment, by 78% in robusta and 92.3% in arabica. Arabica coffee contains pyrazines in lower concentrations than robusta. The presence of 2‐ethyl‐5‐methylpyrazine was observed in both coffee varieties (approximately 4%), but the content decreased significantly following the application of a heating pretreatment. The lower levels of pyrazines contribute to arabica's more delicate and complex flavor profile, which can include floral, fruity, and sweet notes. Pyrazines are formed through the Maillard reaction and Strecker degradation. The Maillard reaction is a chemical process that occurs between amino acids and reducing sugars, resulting in the formation of a variety of flavor and aroma compounds, including aldehydes (Freitas et al. 2024).

The content of furfural was higher in arabica coffee beans (19.25%) than in robusta beans (4.68%). This contributes to the sweeter and more complex flavor profile of arabica coffee, enhancing its characteristic notes of fruit, flowers, and caramel. This compound is formed during the roasting process and has distinct sensory characteristics. Furfural is typically formed from the breakdown of pentoses (five‐carbon sugars) during the roasting of coffee beans. Additionally, it can be generated through the oxidation of furfuryl alcohol. The high temperatures associated with roasting facilitate these chemical reactions (Moon and Shibamoto 2009). 2‐Furanmethanol was present in high concentrations in arabica (7.07%) and in lower concentrations in robusta. A lower concentration of (2‐furanyl)‐1‐ethanone was observed in both varieties. 5‐Methyl‐2‐furancarboxaldehyde (5‐MFC) was identified in both varieties, 6.76% in robusta and 7.42% in arabica. According to the literature, the concentration of 5‐MFC varies depending on the roasting process and gives the coffee its sweet, caramel, and bread‐like notes (Colzi et al. 2017). The differences between arabica and robusta are the abundance of pyrazines (36%), ketones (5.4%), and furans (37.8%) in arabica and the high content of pyrrole (9.25%), pyrazine (35%), and pyridine (24%) derivatives in robusta. The volatile compounds found in arabica are responsible for sweet, caramelized, buttery, and nutty notes, while those found in robusta provide a spicy aroma (Colzi et al. 2017).

The results demonstrate that arabica coffees exhibit a sweeter, softer flavor profile with caramelized and nutty notes, while robusta coffees have a stronger, more bitter flavor with earthy, woody, and spicy notes. These differences are largely attributed to the distinct composition of volatile compounds present in each type of coffee.

## 4 Conclusions

In this study, the content of metals (Na, Mg, K, Ca, P, S, Fe, Mn, Cu, Zn, Cr, Ni, Cd, and Pb) fatty acids profile, and volatile compounds in roasted arabica and robusta coffee were analyzed. Additionally, the concentrations of mineral elements and the leaching percentages in the coffee brews were analyzed and the effects of boiling time were evaluated. The nutritional quality of coffee brews was assessed based on the fatty acids profile using various analytical indices, including the thrombogenic index, the atherogenic index, the hypocholesterolemic/hypercholesterolemic ratio, the health‐promoting index, and the nutritive value index. The atherogenic index values indicate a potential for atherogenesis (formation of fatty deposits in the arteries). In general, the arabica type showed a more favorable lipid profile for cardiovascular health as well as overall nutritive value. The leaching percentage varied according to the specific element, and the leaching degree increased with the boiling time. In general, the major elements showed higher leachabilities than the elements found in trace levels. The concentrations of PTEs in coffee brews were compared with the MALs for drinking water. Except for Mn, the concentrations of all other PTEs were much lower than the corresponding MALs. The health risk assessment demonstrated that PTEs content posed no health risk to consumers exposed at the considered daily coffee ingestion. On the other hand, although the contribution of coffee to the recommended daily intake of essential elements was low, due to its significant daily consumption, it can be considered as a source of essential elements.

The volatile compounds, which are responsible for the flavor profile of coffee, include ketones, pyrazine, pyridine, aldehydes, hydrocarbons, amines, esters, furans, and acids. A total of 61 volatile compounds were identified in the two studied coffee varieties. In general, the arabica variety contains pyrazines in lower concentrations than robusta. The most prevalent pyrazine compound present in elevated concentrations in both varieties was 2‐methylpyrazine, which contributed to the flavor and aroma. The concentration of furfural was found to be higher in arabica than in robusta, contributing to the aroma with notes of sweet caramel and nuts.

## Author Contributions

**Marin Senila:** conceptualization (lead), data curation (lead), funding acquisition (lead), investigation (equal), methodology (equal), supervision (lead), writing – original draft (lead). **Eniko Kovacs:** investigation (equal), writing – review and editing (lead). **Lacrimioara Senila:** formal analysis (equal), investigation (equal), software (lead), writing – original draft (supporting).

## Ethics Statement

The authors have nothing to report.

## Conflicts of Interest

The authors declare no conflicts of interest.

## Data Availability

The data that support the findings of this study are available on request from the corresponding author.
